# Determination of cut-off points for the Move4 accelerometer and assessment of energy expenditure in children and adolescents aged 6–16 years using manual wheelchairs: a validation and calibration study

**DOI:** 10.1186/s12984-026-02085-5

**Published:** 2026-07-02

**Authors:** Selina Seemüller, Anne Kerstin Reimers, Benedikt Meixner, Florian Engel, Franziska Beck

**Affiliations:** 1 Department of Sport Science and Sport, Friedrich-Alexander-Universität Erlangen-Nürnberg, Erlangen, Germany; 2 Integrative and Experimental Exercise Science & Training, Julius-Maximilians-Universität Würzburg, Würzburg, Germany

## Abstract

**Background:**

The present study aimed to determine activity intensity and validate the Move4 accelerometer and derive cut-off points to accurately classify wheelchair-based physical activity (PA) in children and adolescents.

**Methods:**

A calibration and validation study with a test–retest design was performed in 24 children and adolescents (mean age 9.9 ± 3.2 years; 50% girls) using manual wheelchairs. Participants used a manual wheelchair as their primary mode of mobility and were able propelling the wheelchair independently. Participants completed standardized activities while wearing Move4 sensors at the wrist, upper arm, and chest. In a subsample of 7 children and adolescents, energy expenditure was recorded using portable metabolic cart. Mean Amplitude Deviation (MAD) and Movement Acceleration Intensity (MAI) were processed to derive cut-off points for sedentary, light, moderate, and vigorous PA using CART models, and validity was assessed via Metabolic Equivalent (MET) and heart-rate responses.

**Results:**

Both MAD and MAI values increased systematically across activity intensities, supporting good criterion validity. MET values showed progression from sedentary to vigorous tasks. Intensity-specific cut-off points were successfully established for all sensor placements, with consistently higher thresholds for MAI. Sensitivity and specificity analyses indicated the most balanced performance for the upper arm (MAD) and chest (MAI). Test–retest comparisons showed greater variability for MAD-based cut-offs, whereas MAI thresholds demonstrated higher stability, with deviations of approximately 4–5%.

**Conclusions:**

While both MAD and MAI validly reflected PA intensity, MAI showed greater temporal stability and test–retest robustness. These findings support the use of accelerometery for classifying PA in children and adolescents using wheelchairs and underscore the importance of selecting appropriate metrics and sensor positions.

## Introduction

Children with disabilities have unique needs, particularly when it comes to physical activity (PA) [1]. Children and Adolescents using wheelchairs (CAUW) are often unable to participate in different types of PA due to social, individual, or environmental barriers [2]. Unlike their peers without disabilities, they often face challenges requiring adapted approaches to ensure their participation in a physically active lifestyle (Jaarsma et al., 2014). An adequate level of PA is crucial for the physical, emotional, and social well-being of CAUW (Seemüller et al., 2023). Further being physically active reduces the risk of overweight, lower life satisfaction and low vitality [3–6]. However, to benefit from the beneficial health effects of PA, the World Health Organization (WHO) developed PA-guidelines (Bull et al., 2020). Accordingly, CAUW should be active for an average of at least 60 min per day across the week in moderate-to-vigorous PA (MVPA), with an emphasis on aerobic activity [7]. Due to the health benefits of PA, it is important to have device-based measurement methods to assess the amount and intensity of PA. CAUW have different motion sequences than children who can walk. Their PA is primarily achieved by propelling the wheelchair with the upper limbs. Consequently, reliable and valid instruments are required to accurately assess PA levels in CAUW [8–10].

In general, there are two options measuring PA in children: subjective as well as device-based measurement methods [11–13]. Subjective measurement methods are mostly retrospective questionnaires, interviews or PA diaries. These are inexpensive and provide useful evidence on the context and on the subjective perception of the participants’ physical behavior. However, subjective measurement methods are lower in accuracy when quantifying amount and intensity of PA when compared to device-based measurement methods [11]. In research involving CAUW, interviews are most commonly used, as they do not require adaptation. Questionnaires need to be adapted in terminology as well as extended due to other PA settings, such as movement related therapy [14]. Device-based measurement methods of PA involve device-based tools like motion sensors (e.g., accelerometers, pedometers, and heart rate monitors) and calorimetry [13]. These device-based measurement methods provide an objective and non-invasive way to assess PA because they capture movement continuously and independently of participant recall, thereby improving measurement validity and reliability compared to self-report measures [15]. Moreover, device-based methods allow for a more accurate quantification of PA intensity by capturing movement dynamics and acceleration patterns across different intensity levels. Further, they are especially valuable for younger children as they often struggle to accurately recall or estimate their past PA, and their intermittent activity patterns make proxy reports particularly challenging [16, 17].

Building on the advantages of device-based PA assessment for accurately quantifying intensity levels, accelerometers and pedometers are cost-effective and simpler alternatives to calorimetry and double-labeled water, offering objective assessments of PA while avoiding recall bias and social desirability [18]. However, pedometers, which are measuring steps using a mechanical sensor, are not applicable in individuals using wheelchairs. Accelerometers provide more comprehensive data by tracking movement acceleration in multiple planes, allowing an exact analysis of duration, intensity and frequency of PA [19, 20]. Importantly, accelerometer outputs are highly influenced by sensor placement [21]. In wheelchair users, upper-limb–dominated propulsion and limited trunk movement may result in substantially different acceleration patterns across body locations, making it necessary to evaluate and compare multiple sensor positions when deriving PA intensity cut-off points.

For the meaningful application of accelerometers, calibration and validation studies are required because raw acceleration signals do not directly reflect energy expenditure or PA intensity and are influenced by factors such as age, body size, movement patterns, and activity type. Therefore, population-specific thresholds are necessary to ensure valid and reliable quantification of PA duration and intensity [22, 23]. Thereby accelerometer devices from different brands (e.g., Actigraph, Movisens, Vitamove) are used in studies, often relying on statistical models to estimate intensity levels. These approaches often require reference methods such as indirect calorimetry, although these methods are expensive, time-consuming, and impractical for free-living conditions, especially for children [24, 25].

Separate cut-off points are needed for CAUW because standard thresholds (for light, moderate, and vigorous activity) are based on walking patterns and energy expenditure of children without disabilities. The sensor must be validated to ensure accurate measurement of PA intensity in this population. In this context, Nooijen, de Groot [26] validated an activity monitor (VitaMove) for older CAUW (> 10 years). In addition to this, a more recent study has examined the validity of wearable activity sensors in CAUW by evaluating their ability to classify broad activity categories such as stationary and locomotion under naturalistic conditions [27]. While this work provides valuable evidence that movement patterns of pediatric wheelchair users can be detected reliably using accelerometery, it did not address intensity-related outcomes or the calibration of metabolic equivalent (MET)-based cut-off points. Therefore, despite emerging research on activity classification in CAUW, there remains a lack of studies establishing intensity-specific thresholds for accelerometers in this population. Thus, we aimed to determine activity intensity cut-off points and validate an accelerometer for CAUW. This sensor (Move4, movisens GmbH, Karlsruhe, Germany) has already been extensively validated for adults as well as children without disabilities [28–30].

Therefore, the research question addressed in this study was the determination of activity intensity cut-off points for the Move4 accelerometer for CAUW aged 6 to 16 years. Specifically, we outlined the following three objectives:

1. Validation of selected activities for the PA levels (sedentary behavior (SB), light PA (LPA), moderate PA (MPA), vigorous PA (VPA)) by using heart rate as well as the metabolic equivalent (MET) measured with a metabolic cart.
2. Modelling and determination of cut-off points to distinguish different activity levels (SB, LPA, MPA, VPA).

- by using different metrics: mean amplitude deviation (MAD) [31] and movement acceleration intensity (MAI) [32].
- by differing between the three sensor positions (upper arm, wrist, chest) for mapping individual movement activities representation of cut-off points per movement activity per position.

3. Determination of the test-retest agreement by using data from two measuring time points to determine the test-retest reliability of the Move4 together with the respective metrics (MAD and MAI).

## Methods

### Study design

The present study was designed as a laboratory-based calibration and validation study with a test–retest design to derive accelerometer-based cut-off points for PA intensity levels in CAUW. Participants completed a standardized protocol consisting of six wheelchair-based activities covering SB, LPA, MPA and VPA in two measurement sessions separated by one week. During each session, raw acceleration data were collected using the Move4 sensor at three body positions, while energy expenditure was assessed using portable metabolic cart in a subsample of 7 children and heart rate was continuously recorded. The study was approved by the local Ethics Committee (Ref. No. 24–345-S) and was therefore in accordance with the 1964 Declaration of Helsinki [33]. Eligible children and their parents or legal guardians received written information about the study procedures and potential risks. Written informed consent was obtained from both the child and at least one parent or legal guardian prior to participation. Data collection took place from January to March 2025. The study ended as planned after completion of data collection. No changes to the study methods or eligibility criteria occurred after study commencement.

### Participants

This study included male and female CAUW aged 6 to 16 years, mean age 9.9 ± 3.2 years, for details see Table 2. 24 participants were recruited through the VAMED clinic in Hohenstücken, Brandenburg, Germany, a specialized rehabilitation centre for children and adolescents with spinal cord injury and related mobility impairments, as well as via sports clubs and personal contacts across Germany. Children recruited through the clinic were not required to be in inpatient care at the time of participation; measurements were conducted independently of treatment status. All participants were regular wheelchair users in daily life.

Inclusion criteria were:

(i) age between 6 and 16 years,
(ii) regular use of a manual wheelchair as the primary mode of mobility, and.
(iii) the physical ability to perform upper-body exercise tasks such as wheelchair propulsion at varying intensities.

Exclusion criteria were:

(i) acute medical conditions or contraindications that could prevent safe participation in PA (e.g., acute infections, uncontrolled cardiovascular conditions),
(ii) multiple disabilities that could impair the understanding or execution of the standardized activity protocol (e.g., severe cognitive impairments), and.
(iii) any additional medical condition that could substantially affect movement execution or physiological responses beyond the limitations inherent to wheelchair use.

As this study aimed to determine accelerometer cut-off points in a methodological calibration framework, no formal a priori power calculation was conducted. Instead, the sample size was guided by previous accelerometer calibration studies in pediatric populations and wheelchair users, which commonly included comparable sample sizes [23, 26].

### Procedures

The study was designed as a test–retest study to assess the validity and consistency of the cut-off points between the first (T1) and second (T2) measurement occasions, separated by one week. Data collection was pseudonymous, with each participant assigned a unique personal code consisting of letters and numbers. Additionally, each child’s weight and height were asked from the children, and their age was documented. The children’s fitness level was assessed using the question: “How many hours of exercise do you do per week?”. Although this single-item measure is not a psychometrically validated fitness test, similar weekly duration questions are widely used in epidemiological PA surveys to approximate habitual PA levels and provide contextual information on participants’ typical engagement in exercise [34].

### Determination of the Move4 accelerometer cut-off points for children

To determine accelerometer cut-off points for the Move4 sensor and to confirm the expected MET-values for CAUW, participants performed six different standardized activities in the wheelchair under the supervision of the trained research team. Predefined speeds were selected instead of self-paced intensities to ensure standardized external workload across participants, thereby improving comparability of accelerometer outputs and physiological responses for cut-off point determination. Although relative exercise intensity may have varied between participants, particularly according to age and physical capacity, standardized pacing was considered necessary to reduce variability in movement execution. The selected activities included reading in a seated position, being pushed in the wheelchair, slow self-propelled wheeling (2 km·h^− 1^), moderate paced self-propelled wheeling (4 km·h^− 1^), self-propelled wheeling a ramp up and down (inline 6%), fast wheeling (8 km·h^− 1^). The selected activities covered four intensity levels: SB, LPA, MPA, and VPA. The activity protocol was designed to reflect common wheelchair-based movements performed in daily life and sport-related contexts among CAUW, while covering a broad range of PA intensities. The use of a structured activity protocol with predefined movement tasks and externally controlled intensity levels is consistent with established procedures in accelerometer calibration studies and is considered necessary for the derivation of device-specific activity intensity cut-off points [30, 35]. Activities were selected based on recommendations for accelerometer calibration studies emphasizing ecological validity and representative movement patterns [36, 37]. In addition, expected intensity levels were informed by both the Youth Compendium of Physical Activity and the Adult Wheelchair Compendium of Physical Activity, as no pediatric wheelchair-specific compendium currently exists [38, 39]. Table 1 provides an overview of the selected activities and their corresponding expected metabolic equivalent (MET) values.

**Table 1:** Activity level and expected MET values

Activity level	Activity	MET in adult wheelchair compendium [40]	MET in youth compendium of physical activities (6–16 years) [38]	Expected MET-values
Sedentary behavior (1.0 −1.5 MET)	Reading	1.2	1.3	1.3
	Being pushed	1.1	Not available	1.1
Light PA (1.5–2.9 MET)	Slow wheeling (2 km·h^− 1^)	2.4	2.6 (walking 2 km·h^− 1^)	2.5
Moderate PA (3.0–6.0 MET)	Moderate wheeling (4 km·h^− 1^)	3.8	3.5 (walking 4 km·h^− 1^)	3.6
	Wheeling up and down a ramp (6%)^1^	4.0	Not available	4.0
Intense PA (> 6 MET)	Fast wheeling (8 km·h^− 1^)^2^	5.9	7.4 (walking 8 km·h^− 1^)	6.6

^1^This ramp is already structurally installed in the clinic and complies with the DIN 18040-1 standard

^2^During this exercise, propulsion is not performed continuously for four minutes. Instead, it is divided into intervals of 45 s of activity followed by 15 s of rest

**Fig. 1:**
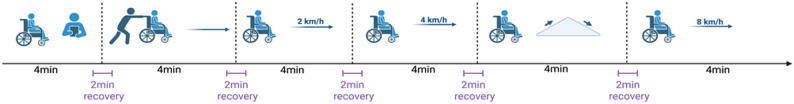
Testing process. Created in BioRender. Meixner, B. (2026) https://BioRender.com/bc45lb3

Each activity was performed for a duration of four minutes. The wheeling activities were performed in an indoor gymnasium on a predefined oval track. Participants wheeled continuously in a clockwise direction along the marked circuit, allowing uninterrupted propulsion during each activity bout. The course design enabled standardized speed control while minimizing abrupt stopping, except at designated transition points between activities. Figure 1 shows the testing process in detail.

For the fast wheeling task (8 km·h⁻¹), continuous propulsion over the full four-minute period was not feasible for all participants. Therefore, this activity was performed in an intermittent format consisting of 45 s of active propulsion followed by 15 s of passive recovery, repeated throughout the four-minute period. This approach was chosen to ensure feasibility and safety while maintaining a high-intensity workload representing vigorous PA. Data collection took place during a 60-min session, consisting of approximately 40 min of structured activity testing and 20 min allocated to preparation and follow-up procedures (e.g., sensor placement, instructing participants, consent confirmation, and device removal). Before starting the test, verbal consent was obtained again from each child. Each child could give consent separately for each sensor as well as for the portable metabolic cart. The research team applied the sensors to the different body parts of the participants: wrist/non-dominant hand (Move4), upper arm/non dominant (Move4) and chest (in combination with ECG) (EcgMove4). In addition, a portable metabolic cart was used in participants who consented to wearing the device.

Each activity lasted four minutes. A research team member guided the children through each activity and set the pace for wheeling exercises to help them maintain a consistent speed. Therefore, a predefined distance (2 km·h^− 1^: 33 m per minute; 4 km·h^− 1^: 66 m per minute; 8 km·h^− 1^: 133 m per minute) as well as a stopwatch was used to estimate speed. Throughout the session, the research team monitored the participants to ensure proper execution of each activity. A two-minute recovery period was provided between activities to allow for smooth transitions. The duration of the recovery period was informed by pilot testing conducted prior to the study, indicating feasibility within the target population while maintaining the overall testing procedure. During the first minute of this recovery period, children were instructed to sit still to ensure clear separation between activity segments in the accelerometer data.

Once all six activities were completed, the sensors as well as the metabolic cart were removed. Although a two-minute rest period was provided between activities, it is assumed that heart rate may not return to resting levels after moderate and high-intensity exercises. To account for this physiological carry-over, the sequence of activities was therefore structured from low to high intensity, minimizing potential confounding effects on subsequent measurements.

### Measurement instruments

The movisens Move4 (movisens GmbH, Karlsruhe, Germany) is a wearable sensor developed to track PA and sleep. The EcgMove4 (movisens GmbH, Karlsruhe, Germany), which includes the same motion-sensing capabilities as the Move4 (i.e., assessment of PA and sleep), also records a single-channel ECG, which enables to measure heart rate and heart rate variability. Both devices integrate a 3-axis accelerometer and a 3-axis gyroscope, allowing for accurate detection of movement patterns and body posture.

All collected data is saved on internal flash memory and can later be processed with the movisens Data Analyzer software. This software provides a variety of analysis techniques and visualization options to interpret the data, offering detailed insights into PA, sleep behavior, heart rate and heart rate variability. It can generate key outcome measures such as activity classifications, posture recognition, step count, energy expenditure, and metabolic equivalents. No changes to outcome measures occurred after study commencement.

#### Respiratory gas analysis

In a subsample of 7 children, respiratory gas exchange was continuously monitored during six activities using a portable, open-circuit, breath-by-breath metabolic system (MetaMax 3B; Cortex Biophysik GmbH, Leipzig, Germany). The MetaMax 3B was calibrated before each test using high-precision reference gases (15% O_2_, 5% CO_2;_ Cortex Biophysik GmbH, Leipzig, Germany) and a 3-L calibration syringe.

The system measures expired ventilation (V̇_E) as well as oxygen uptake (V̇O₂) and carbon dioxide output (V̇CO₂) on a breath-by-breath basis during rest and PA. Respiratory flow is assessed using a bidirectional mechanical turbine wheel, while the fractions of O₂ and CO₂ in expired air are analyzed via electrochemical and infrared sensors, respectively. Breath-by-breath V̇O₂ and V̇CO₂ are calculated using standard metabolic algorithms, including the Haldane transformation, allowing for continuous estimation of energy expenditure.

Metabolic equivalents (METs) and energy expenditure were derived from gas exchange data according to established indirect calorimetry equations, by expressing V̇O₂ relative to body mass and resting metabolic rate (41). MET values were calculated by relating measured V̇O₂ to resting metabolic rate (1 MET ≈ 3.5 mL·kg⁻¹·min⁻¹), enabling comparability of activity intensity across tasks and populations [42].

The MetaMax 3B has previously demonstrated reliable and valid measurements during rest as well as during MPA and VPA in adolescents [41].

### Data processing

Data processing consisted of two main steps: data preparation and metric computation. Data preparation was performed using MATLAB (The MathWorks, Natick, MA, USA; version R2025b). Only basic MATLAB functions were used for data trimming, synchronization, and annotation; no specialized toolboxes were required. The sequence of activities was imported to enable annotation of both the individual activities and the breaks between them within the sensor data. These annotations served as a reference for assigning the calculated PA metrics to the corresponding activities. Additional parameters, including measurement time point, sex, age, and heart rate, were integrated into the dataset.

For metric computation, accelerometer data were processed using a combination of MATLAB and the movisens DataAnalyzer software. The proprietary algorithms implemented in DataAnalyzer were used to compute the MAD and MAI metrics.

Spirometric data were analyzed using the device’s proprietary software to obtain metabolic equivalent (MET) values. For each participant and activity, the mean MET value over the stable 4-min interval was calculated and used as the physiological reference measure.

### Statistical analysis

Statistical analyses were conducted using MATLAB. Accelerometer cut-off points for the four activity intensity levels (SB, LPA, MPA, VPA) were derived using a decision tree approach based on Classification and Regression Trees (CART) (MathWorks, 2025). This method applies recursive partitioning to split the dataset until a predefined stopping criterion (maximum tree depth) is reached, aiming to reduce variance and maximize information gain.

Cut-off points were determined for the overall sample as well as separately by sex and by measurement time point. Sensitivity and specificity were calculated to evaluate the classification performance of the derived cut-off points.

To assess criterion validity, a descriptive approach was applied using three complementary criteria: [1] the expected increase in accelerometer-derived acceleration values (MAD and MAI) across activity intensities [2], physiological responses reflected by heart rate, and [3] MET values obtained from the portable metabolic cart. Criterion validity was evaluated by examining whether accelerometer-derived values increased consistently across predefined activity intensity levels and corresponded with expected physiological responses. Test–retest reliability was examined by comparing cut-off points derived at the two measurement time points. Descriptive analyses included the generation of boxplots to visualize heart rate distributions across activities.

## Results

### Sociodemographic data

Accelerometer-based analyses were conducted in all participants (*n* = 24), whereas MET analyses were based on the subsample (*n* = 7). The average amount of weekly sports activity was 270 min (SD = 110.6), with a range between 120 and 480 min per day. Detailed sociodemographic data can be seen in Table 2.

**Table 2:** Anthropometric characteristics of the study sample

	Overall	Boys	Girls
Accelerometers			
N (%)	24	12 (50)	12 (50)
Age [years; M ± SD]	9.9 (3.2)	10 (3.6)	9.8 (3.0)
Weight [kg; M ± SD]	36.3 (17.5)	41.2 (19.5)	31.6 (15.6)
Height [cm; M ± SD]	137.3 (23.1)	137.3 (23.5)	137.4 (20.5)
Body Mass Index (BMI) [kg*m − 2] [M (range)]	20.11 (10.4–32.6)	19.4 (10.4–32.6)	21.8 (14.8–29.1)
Sports activity [min/week] [M (range)]	270 (120–480)	241.3 (120–375)	280 (135–480)
Measurements with Portable Metabolic Cart in a Subsample			
N (%)	7 (100)	3 (42.9)	4 (57.1)
Age [years; M (range)]	12.8 (10–16)	14.0 (13–15)	12.0 (10–16)
Weight [kg; M (range)]	44.7 (21–61)	53.3 (48–61)	38.3 (21–55)
Height [cm; M (range)]	155 (118–181)	162.3 (151–181)	149.5 (128–174)
Body Mass Index (BMI) [kg*m − 2] [M (range)]	18.2 (12.8–22.4)	20.3 (18.6–22.4)	16.6 (12.8–20.1)
Sports activity [min/week] [M (range)]	285.0 (120–480)	280 (120–480)	288.8 (135–360)

Values are presented descriptively. No statistical comparisons between boys and girls were performed due to the small sample size and the descriptive purpose of the table

Missing data were present for some analyses. While all 24 participants completed the accelerometer protocol, metabolic data were available only for a subsample of seven children due to the limited feasibility of spiroergometric testing and participant tolerance. In addition, segments of accelerometer data were excluded during preprocessing when activities could not be performed continuously for the full four-minute duration, particularly during high-intensity wheeling tasks. Analyses were performed using available data for each activity, resulting in varying sample sizes across specific analyses. Although this approach may reduce statistical power and limit generalizability, it allows for accurate representation of the measured activities.

### Mean accelerometer and metabolic values across activities

#### Acceleration values measured by accelerometer

The accelerometer outcomes for each activity differentiated by the MAD and MAI metrics at the wrist, upper arm, and chest positions are presented in Table 3. Overall, MAI values were consistently higher than MAD values across all activities and sensor placements. The lowest mean accelerometer values were observed during reading, whereas the highest values occurred during high-intensity wheeling activities, particularly wheeling at 8 km·h^− 1^ and ramp wheeling. Both metrics generally increased with increasing activity intensity across all sensor locations. Overall, the highest acceleration values were observed at the wrist, followed by the upper arm, with the lowest values recorded at the chest.

**Table 3:** MAD and MAI metrics accelerations (mean absolute deviation, mg) in the six activities

	Wrist		Upper arm		Chest	
	MAD	MAI	MAD	MAI	MAD	MAI
Activity	Mean (SD)	Mean (SD)	Mean (SD)	Mean (SD)	Mean (SD)	Mean (SD)
Reading	14.6 (16.0)	50.9 (53.1)	11.1 (15.7)	25.8 (27.3)	7.1 (4.2)	18.1 (11.4)
Being pushed	33.7 (16.8)	80.2 (52.5)	28.0 (13.8)	57.0 (25.6)	20.6 (6.2)	44.2 (17.1)
Wheeling 2 km·h^− 1^	174.4 (64.7)	373.9 (121.6)	98.8 (41.1)	215.7 (70.2)	38.6 (20.3)	96.5 (54.5)
Wheeling 4 km·h^− 1^	340.3 (149.3)	594.4 (214.8)	182.1 (89.1)	346.9 (149.4)	82.8 (42.2)	188.5 (96.2)
Wheeling a ramp up and down	305.9 (159.3)	527.2 (234.6)	287.8 (147.5)	331.8 (143.6)	90.5 (39.1)	204.7 (85.0)
Wheeling 8 km·h^− 1^	515.7 (248.8)	833.4 (345.5)	166.5 (152.4)	506.4 (233.4)	131.7 (62.5)	279.2 (108.2)

#### MET values measured by metabolic cart

Table 4 presents the mean MET values derived from the metabolic cart compared to the expected reference ranges [38, 39]. With the exception of sedentary behavior (SB), all calculated MET values fell within their respective expected ranges for LPA, MPA and VPA. However, all activity categories showed slightly higher MET values than the reference averages. Specifically, the mean MET value for SB activities (reading, being pushed) was 2.1, which is above the reference range of 1.0–1.5.

**Table 4:** MET-values of the portable metabolic cart

Activity level	Activity	Expected MET-values (38, 39)	Average MET-values
Sedentary behavior (1.0 −1.5 MET)	Reading	1.3	2.0
	Being pushed	1.1	2.1
Light PA (1.5–2.9 MET)	Slow wheeling (2 km·h^− 1^)	2.5	2.9
Moderate PA (3.0–6.0 MET)	Moderate wheeling (4 km·h^− 1^)	3.6	4.3
	Wheeling up and down a ramp (6%)^1^	4.0	4.7
Vigorous PA (> 6 MET)	Fast wheeling (8 km·h^− 1^)^2^	6.6	7.3

### Criterion validity

Criterion validity was assessed using three complementary criteria: [1] the expected increase in accelerometer-derived acceleration values (MAD and MAI) across activity intensities [2], physiological responses reflected by heart rate, and [3] MET obtained from portable metabolic cart. First, criterion validity was assessed descriptively by examining mean acceleration values (MAD and MAI, in mg) across the different activities for the three sensor positions (wrist, upper arm, chest). As expected, the acceleration values increased with higher activity intensities, with the lowest values observed during sedentary behavior such as reading, and the highest values recorded during wheeling at higher speeds (e.g., 8 km·h^− 1^) or when wheeling up and down a ramp. In line with the physiological load of the tasks, both MAD and MAI metrics showed a gradual stepwise increase from light to vigorous activities. Across all conditions, MAI values were consistently higher than MAD values, indicating that the MAI metric is more sensitive to variations in movement intensity. The wrist placement generally produced the highest acceleration outputs, while the chest sensor yielded lower absolute values, particularly during sedentary and light activities.

As a second criterion, heart rate was used as an independent physiological reference to evaluate whether accelerometer-derived activity intensities corresponded to cardiovascular load.

**Fig. 2:**
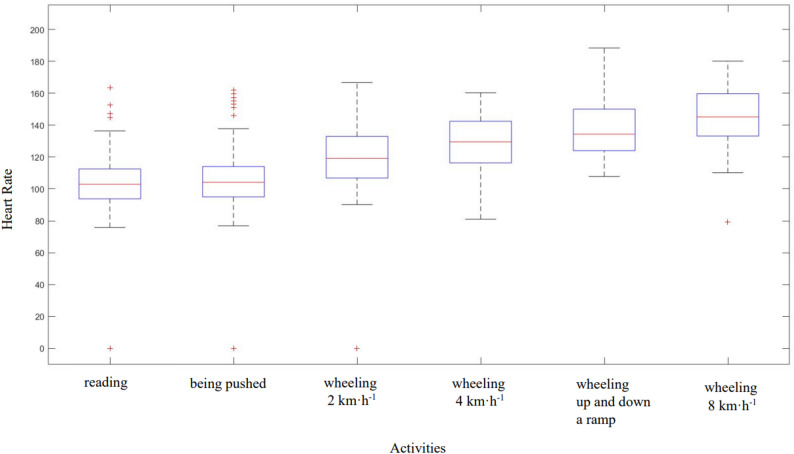
Intensity of heart rates during individual activities

The mean heart rate increased progressively with activity intensity, averaging 113 beats (SD = 34) per minute (bpm) during SB, 121 bpm (SD = 25) for LPA, 133 bpm (SD = 18) for MPA, and reaching the highest values during VPA with a mean of 144 bpm (SD = 21). This stepwise increase reflects the expected cardiovascular response to increasing physical demands. The mean correlation coefficient between acceleration values and heart rate was 0.605 (*p* < 0.01) for the MAD metric and 0.595 (*p* < 0.001) for the MAI metric, indicating a strong correlation for both MAD and MAI metric. Figure 2 presents each activity with the associated heart rate.

As a third criterion of validity, MET obtained from portable metabolic cart were used to verify whether the selected activities corresponded to the predefined intensity categories (SB, LPA, MPA, VPA) assumed during protocol development. As expected, MET values increased progressively with higher activity demands—from sedentary behaviors such as reading to more vigorous tasks like fast wheeling or ramp wheeling (see Table 4). Importantly, the mean MET values of the individual activities fell within or close to the expected MET ranges defined for their respective intensity levels based on the Youth Compendium of PA [38] and the Adult Wheelchair Compendium [40]. This finding confirms that the selected activities were appropriately assigned to their intended intensity categories and therefore supports the validity of the activity protocol used for accelerometer calibration. This pattern supports the validity of the spirometry data in reflecting physiological load and confirms its suitability for verifying the classification of activity intensity.

### Cut-off points of intensity levels/thresholds

Based on the performed activities, three cut-off points were derived to distinguish between SB, LPA, MPA, and VPA using both the MAD and MAI metrics (see Table 3). The results indicate that valid thresholds could be established for all three sensor positions (upper arm, wrist, chest).

Overall, cut-off values differed systematically between MAD and MAI, with consistently higher thresholds observed for the MAI metric across all sensor positions (see Table 5). For example, the transition from MPA to VPA was identified at 212.1 mg (MAD) vs. 395.0 mg (MAI) for the upper arm, 387.3 mg vs. 652.0 mg at the wrist, and 101.7 mg vs. 224.1 mg at the chest. Similarly, the cut-off points separating SB from LPA and LPA from MPA were consistently higher for the MAI compared to the MAD metric across all sensor locations.

No meaningful descriptive differences in accelerometer-derived cut-off points were observed between boys and girls across sensor positions and metrics. Given the exploratory nature of the study and the relatively small sample size, sex-specific inferential analyses were not considered sufficiently robust. Therefore, overall cut-off points are reported. These findings confirm that while the relative activity thresholds are consistent across sensor placements, the absolute cut-off values vary by both metric and sensor location, with the wrist yielding the highest thresholds.

**Table 5:** Overall cut-off points with MAD and MAI metrics (mg)

	Cut 1 (SB-LPA)		Cut 2 (LPA-MPA)		Cut 3 (MPA-VPA)	
Accelerometer position	MAD	MAI	MAD	MAI	MAD	MAI
Upper arm	46.0	99.5	189.6	298.1	212.1	395.0
Wrist	74.2	168.3	273.5	498.8	387.3	652.0
Chest	22.1	52.9	70.6	163.2	101.7	224.1

### Sensitivity and specificity

Table 6 summarizes the sensitivity and specificity values for the classification of PA intensities across sensor placements and both metrics (MAD and MAI). For SB, the wrist-worn accelerometer showed the highest sensitivity for both MAD (98.7%) and MAI (98.7%), while specificity was highest for the upper arm in MAD (96.7%) and for the chest in MAI (94.9%).

Overall, the upper-arm sensor provided the most balanced performance for the MAD metric, showing a good trade-off between sensitivity (64.7%) and specificity (77.1%). For the MAI metric, the chest sensor achieved slightly higher specificity (75.2%) while maintaining a comparable level of sensitivity (59.2%).

**Table 6:** Sensitivity and specificity of cut-off points (%) (MAD and MAI metrics)

Accelerometer position	SB		LPA		MPA		VPA		Overall	
	Sens.	Spec.	Sens.	Spec.	Sens.	Spec.	Sens.	Spec.	Sens.	Spec.
MAD metric										
Upper Arm	93.2	96.7	75.3	67.3	45.8	54.0	44.7	90.6	64.7	77.1
Wrist	98.7	84.9	78.4	57.2	35.7	51.5	17.8	98.6	57.7	73.1
Chest	81.6	94.0	28.1	73.5	50.2	56.6	67.4	78.8	56.8	75.7
MAI metric										
Upper Arm	91.7	95.9	85.2	67.2	37.5	54.5	50.4	86.8	66.2	76.1
Wrist	98.7	82.1	68.6	55.9	24.8	53.0	34.1	94.0	56.6	71.2
Chest	80.5	94.9	41.7	73.3	41.7	57.0	73.1	76.0	59.2	75.2

### Test–retest agreement

Given the test–retest design of our study, cut-off points for the different intensity levels were compared between T1 and T2 across all accelerometer positions. For the MAD metric, differences between the two time points varied depending on sensor position and cut-off level. The largest relative deviations were observed for the SB–LPA threshold at the chest position (13.1%) and for the MPA–VPA threshold at the upper arm (−6.7%). Overall differences across cut-off levels ranged from −15.0% for the upper arm to −2.8% for the wrist, while the chest position showed a positive overall deviation of 42.6% (Table 7). Across cut-off levels, the SB–LPA transition tended to show larger discrepancies compared to the LPA–MPA threshold, which exhibited relatively small differences across all sensor positions.

For the MAI metric, test–retest differences were generally smaller and more consistent across positions and cut-off levels. Overall deviations ranged from −5.8% for the upper arm to 2.6% for the wrist, while the chest position showed a higher overall difference of 26.4%. Across all sensor locations, the SB–LPA threshold again showed the largest relative differences, whereas the LPA–MPA and MPA–VPA cut-offs demonstrated comparatively high stability between T1 and T2.

**Table 7:** Differences between T1 and T2 valued for the MAD and MAI metric (mg)

Accelerometer	Cut 1 (SB-LPA)			Cut 2 (LPA-MPA)			Cut 3 (MPA-VPA)			Overall
	T1	T2	Diff.^1^	T1	T2	Diff^1^	T1	T2	Diff.^1^	Diff.^1^
MAD Metric										
Upper Arm	45.2	44.5	−1.6%	156.3	145.8	−6.7%	221.3	207.0	−6.7%	−15.0%
Wrist	69.5	73.5	5.8%	278.4	264.8	−4.9%	392.7	377.9	−3.8%	−2.8%
Chest	23.5	26.6	13.1%	66.9	77.0	15.1%	96.4	110.4	14.5%	42.6%
Overall			17.3%			3.5%			4.0%	
MAI Metric										
Upper Arm	92.3	95.4	3.2%	303.0	292.0	−3.6%	404.5	383.7	−5.4%	−5.8%
Wrist	150.0	162.9	8.0%	504.3	487.8	−3.3%	656.6	643.0	−2.1%	2.6%
Chest	52.0	57.7	9.7%	158.4	171.6	8.3%	216.7	236.4	8.3%	26.4%
Overall			21.0%			1.4%			0.8%	

^1^*Diff.* Difference

No adverse events or harms were observed during testing. No missing accelerometer data occurred; MET data were missing for participants who declined to wear the metabolic cart.

## Discussion

The primary objective of this study was to determine activity intensity cut-off points and validate the Move4 accelerometer in a sample of CAUW. More specifically, the first aim was to validate the selected activities with regard to activity levels, using both heart rate and METs obtained from a portable metabolic cart as reference measures. The second aim focused on the modelling and determination of cut-off points to differentiate between SB, LPA, MPA, and VPA. For this purpose, two different accelerometer-derived metrics were applied: MAD and MAI. Additionally, three sensor placements (wrist, upper arm, and chest) were analyzed to provide a differentiated representation of activity-specific cut-off points. Finally, a third aim of the study was to examine the test–retest agreement by comparing cut-off points across two measurement time points to assess the reliability of the Move4 and the respective metrics.

### Validity

In this study, the validity of activity classification and intensity assessment was evaluated using a multi-criterion approach, incorporating accelerometer-derived acceleration values (mg), heart rate responses, and metabolic equivalents (MET) obtained from portable metabolic cart. Together, these three criteria allowed for the examination of convergent validity across mechanical, cardiovascular, and metabolic indicators of PA intensity. The MET data served as the physiological criterion to validate the accuracy of the accelerometer-based metrics (MAD and MAI) in distinguishing activity intensity levels [31]. Overall, the results demonstrated a generally consistent increase in MET values across the three of four activity levels (LPA, MPA, VPA), confirming the expected intensity progression and supporting the validity of the accelerometer signals for capturing differences in upper-body activity intensity. This stepwise increase was mirrored by corresponding increases in mean acceleration values and heart rate across the same activities, indicating consistent responses across all three validity criteria. The observed MET value of 2.1 for sedentary activities exceeds the typical reference range of 1.0–1.5 MET proposed by [38] and [40]. Several factors might explain this discrepancy. One possibility is that the CAUW, and were therefore already in a seated position for most activities, which may have influenced energy expenditure measurements and limited variation around the SB threshold [43]. However, this initial assumption needs to be further investigated in studies with larger sample sizes. To date, there is no available evidence regarding energy expenditure in CAUW. Recent research has started to address the paucity of validation studies in pediatric wheelchair users. Engels, Bloemen [27] evaluated the criterion validity of a wearable prototype activity monitor in 37 children using a manual wheelchair. Although this study did not examine energy expenditure or intensity-related outcomes, it provides important evidence that movement-based classification in pediatric wheelchair users is feasible under free-living conditions. The present study extends this evidence by integrating physiological measures, allowing validation not only of activity classification but also of activity intensity. These findings highlight the need for complementary validation approaches that incorporate physiological measures, such as METs or heart rate, to extend classification from activity type to activity intensity.

While acceleration values and heart rate supported the construct and criterion validity of the activity classification, METs measured by indirect calorimetry using a portable metabolic cart were used as the primary reference criterion for modelling the accelerometer cut-off points. The cut-off points derived from the accelerometer data were thus modelled using METs measured with a portable metabolic cart as the criterion measure. This approach follows previous recommendations emphasizing the use of direct metabolic measures rather than heart rate to ensure accurate calibration of accelerometer metrics, particularly in populations with altered cardiovascular responses such as wheelchair users [44, 45]. Based on these findings, cut-off points could be modelled from the accelerometer values. Calibration of accelerometers was conducted in children with an overall normal weight status (mean BMI = 20.1 kg/m²) and relatively high self-reported weekly sports activity (mean = 270 min per week). Nevertheless, variation in activity volume and body composition between participants (see Table 2) likely contributed to differences in heart rate responses during the same activity type [46]. This inter-individual variability should be considered when interpreting the results. Overall, the observed associations and intensity gradients indicate sufficient criterion and construct validity of the Move4 accelerometer for classifying PA intensity in CAUW.

### Selection of activities

The choice of activities in the present calibration study was guided by the need to capture the range of everyday PA typically performed by CAUW. Therefore, the protocol included both activities (e.g., wheeling at different speeds and on a ramp) as well as movement without PA (e.g., being pushed instead of self-propelling). This combination allowed the simulation of various real-life PA patterns and ensured that a broad range of intensity levels and accelerometer outputs could be represented. The selected activities were age-appropriate and reflected movements that are common in daily life for this target group [47]. This approach aligns with best-practice recommendations for calibration studies in children without disabilities [30, 37], emphasizing ecological validity and the inclusion of representative movement types [36]. The initial selection and classification of the activities were based on both the Youth Compendium of PA [38] and the Adult Wheelchair Compendium of PA [40], and were subsequently verified using measured MET values to confirm that the activities corresponded to the intended intensity categories.

Comparable approaches have been applied in previous validation studies involving partly or completely wheelchair-dependent children. For instance, Nooijen, de Groot [48] evaluated the validity of the VitaMove activity monitor in 12 children with spina bifida or cerebral palsy using a structured “wheelchair protocol.” Their activity set included both daily-life and sport-related movements. A similar emphasis on ecologically valid activity selection can be seen in recent work [27]. The study’s activity protocol included a broad range of daily-life and mobility tasks, which were labelled using 5-s video epochs. This approach underscores the importance of including both functionally relevant and context-specific activities when developing accelerometer-based classification models for children with mobility impairments. Our activity selection therefore aimed to balance realism and standardization by including functional movements that reflect the daily experiences of CAUW, while also covering a broad intensity spectrum, from SB (e.g., being pushed) to vigorous, sport-like activities (e.g., fast wheeling). Such comparability strengthens the interpretability of our calibration results and supports the external validity of our findings within the context of existing literature on pediatric wheelchair activity monitoring. However, it should be noted that the number of activities within each intensity category was not perfectly balanced, with fewer light and vigorous-intensity tasks compared to sedentary and moderate activities. A minimum of two activities across each intensity category would strengthen the calibration and classification accuracy of the accelerometer for wheelchair-based movement.

### Cut-off points points

Recent studies emphasize the need to use raw acceleration metrics rather than traditional activity counts to accurately assess PA intensity [45, 49, 50]. Following this recommendation, the present study compared two raw acceleration metrics, MAD and movement MAI, to determine activity intensity cut-off points and validate the Move4 sensor across three sensor positions. In the discussion of results, particular emphasis is placed on the MAD metric, as it is the most commonly used and well-established method for accelerometer calibration [31, 51]. However, the MAI metric was also included, since it applies a band-pass filter that reduces non-movement-related noise and has recently gained attention in validation research [52, 53]. The substantial differences observed between MAD- and MAI-based cut-off points in the present study can be explained by fundamental differences in signal processing. MAD reflects the mean deviation of the raw acceleration signal from its mean and is therefore sensitive to both movement-related accelerations and residual gravitational or postural components. In contrast, MAI applies frequency-based filtering to isolate dynamic movement, thereby reducing non-movement-related signal components. This methodological distinction likely explains why MAI yielded consistently higher cut-off values and demonstrated markedly stronger test–retest stability compared to MAD in the present study.

A comparison of cut-off values can be made with the study by Beck et al. (2023), who used the same sensor (Move4), identical raw acceleration metrics, and comparable sensor placements in children without disabilities. When directly comparing cut-off points, clear differences emerge, particularly for wrist and chest placements. For example, the MAD-based SB–LPA cut-off at the chest was 45.9 mg in ambulant children, whereas substantially lower values were observed in the present CAUW sample. Conversely, wrist-based cut-off values for moderate and vigorous activity tended to be higher in wheelchair users, reflecting continuous upper-body involvement even at lower intensity levels. These findings indicate that, despite identical sensor positions and metrics, absolute cut-off values are not directly transferable between ambulant children and CAUW. This supports the need for population-specific calibration models rather than the application of universal thresholds.

Similar tendencies were observed for the upper-arm and chest placements. Across positions, accelerometer outputs reflected distinct biomechanical demands between populations. While ambulant children predominantly generate acceleration through lower-limb movement, wheelchair propulsion relies on continuous and repetitive upper-body engagement, resulting in altered acceleration profiles even during sedentary or light-intensity activities. These differences may be attributed to distinct biomechanical demands and muscle recruitment patterns, as upper-body propulsion requires greater and more continuous engagement of shoulder and arm musculature [54]. At the same time, the wrist consistently showed the highest acceleration outputs, likely due to its greater mobility and susceptibility to small, non-propulsive arm movements, a pattern also described in able-bodied populations [37, 49, 55]. When comparing the present findings to another validation study in CAUW [26], who used the VitaMove system, the present study determined accelerometer-based intensity thresholds under controlled laboratory conditions using indirect calorimetry as the physiological criterion. Despite these methodological differences, both studies support the validity of accelerometer-based approaches in pediatric wheelchair users and highlight the importance of developing population-specific calibration models rather than applying thresholds derived from ambulant populations.

Furthermore, methodological aspects such as the inclusion of both continuous and intermittent activities in the present protocol may have contributed to more ecologically valid cut-off estimates, as they better reflect the heterogeneous movement patterns typical of wheelchair-based activity. The greater stability observed for MAI-based cut-off points further suggests that this metric may be particularly suitable for defining intensity thresholds in populations with complex and non-cyclic movement patterns. In summary, the observed differences across sensor placements and populations emphasize that both sensor-specific and population-specific calibration are essential to ensure valid and transferable results. The present data therefore provide an important first step toward defining appropriate intensity thresholds for wheelchair-based movement in children and improving the comparability of accelerometer-based assessments in adaptive PA research.

### Sensor positions

To the best of our knowledge, this is one of the first calibration studies in CAUW to compare multiple sensor placements. Because each body position contributes differently to the movement dynamics [56] in different tasks, slight differences in cut-off thresholds across sensor placements were expected. For instance, the MAD cut-offs for SB varied notably across placements: wrist sensors recorded higher acceleration values even during SB compared to chest, which likely reflects involuntary arm movements and sensor sensitivity to arm-based motion. This pattern is consistent with findings in people without disabilites, where wrist-worn accelerometers tend to record higher acceleration values even during low-intensity activities [37].

Beyond SB, similar trends were observed across all intensity levels, with the wrist consistently producing the highest cut-off values for both MAD and MAI metrics, followed by the upper arm and chest. These differences likely result from the greater involvement of the wrist and forearm during propulsion and upper-body movements in CAUW. The chest, being more stable, captured smaller movement amplitudes, especially during light to moderate activity levels. Notably, for VPA, differences between placements became smaller, suggesting that high-intensity wheeling leads to more synchronized movement across upper-body segments.

### Recommendations for sensor positions

The selection of the optimal sensor position should always depend on the study’s primary objective and the specific movement characteristics of the target population. In the present study, we focused on upper-body sensor placements (wrist, upper arm, and chest) since locomotion in CAUW predominantly involves arm and trunk movements rather than lower-limb motion. Among all tested positions, the upper-arm placement demonstrated the highest overall sensitivity and specificity for classifying PA intensity. This result suggests that sensors placed at the upper arm provides a stable signal that captures both propulsion-related and general upper-body movements while minimizing the influence of passive or non-purposeful motions. The upper-arm placement showed good classification performance and may serve as a practical alternative when chest placement is not feasible, for example in free-living settings or when heart-rate measurement is not required. This placement also minimizes interference with daily wheelchair handling. In contrast, the wrist-worn accelerometer exhibited slightly lower specificity and greater susceptibility to movement artifacts caused by non-locomotor arm movements, an issue also reported in studies with children and adults without disabilities [57]. However, its ease of use and higher compliance rates make it an appropriate choice in large-scale or long-term monitoring studies.

Interestingly, our findings are broadly in line with results from studies conducted with adult wheelchair users and paraplegic individuals. For example, a cross-sectional validation study by García-Massó, Serra-Añó [58] examined oxygen consumption (VO₂) and accelerometer signals at multiple body sites in 20 paraplegic adults performing various daily activities (sedentary, propulsion, and housework tasks). They found that the non-dominant wrist placement provided the most accurate estimation of VO₂ (*r* = 0.86), outperforming chest and waist placements. While the optimal sensor position differed in our study—where the upper-arm sensor showed superior classification accuracy, both studies underline that sensor placement substantially influences measurement validity. This comparison highlights an important methodological consideration: optimal sensor positioning may depend on the specific movement patterns, propulsion style, and age-related movement characteristics of the target group.

In adults, wrist movements during wheelchair propulsion tend to be highly rhythmic and tightly coupled to the propulsion cycle [59], making the wrist position of an accelerometer a strong predictor of energy expenditure [44]. In contrast, in children, movements may be more variable and involve additional upper-arm and trunk motion, which could explain why upper-arm placement performed best in our sample.

In summary, upper-arm positions appear most suitable for research requiring precise quantification of PA intensity in CAUW, while the wrist remains useful for feasibility-oriented or ecological monitoring approaches.

### Test-retest agreement

To evaluate the accuracy of the Move4 sensor, a test-retest design was applied to examine the agreement of the cut-off points between T1 and T2 across all sensor positions. Overall, the agreement between measurement occasions was acceptable, with generally small to moderate differences observed between T1 and T2. Among the cut-offs, Cut 3 tended to show larger deviations compared to Cut 1 and Cut 2. This is likely attributable to the greater physiological and motor demands of higher-intensity activities, which are inherently more difficult to perform consistently over a fixed duration, particularly in children. Warner, Vanicek [60] showed that when walking, the values for absolute intensity (e.g. in MET) and relative intensity vary greatly across individuals. This variability is expected to be even more pronounced in very vigorous wheelchair-based activities, which require sustained upper-body effort and precise coordination. In particular, SB activities (such as reading or being pushed) showed minimal variation, whereas maintaining a constant movement pattern during four minutes proved challenging for some children during VPA tasks (e.g., wheeling at 8 km·h^− 1^). Regarding sensor placement, the largest relative differences between T1 and T2 were observed for the upper-arm accelerometer, particularly at the MPA–VPA transition. This finding can be explained by task-specific demands rather than by inferior sensor performance. The four-minute wheeling task at 8 km·h^− 1^ proved challenging for some participants, leading to both inter-individual (between children) and intra-individual (between T1 and T2) variability in upper-arm movement patterns. Accordingly, moderate deviations between measurement occasions were primarily observed at higher intensity levels, where fatigue, pacing strategies, and compensatory movements are more likely to occur. Despite this variability at the highest intensity level, the upper-arm placement showed a favorable balance between sensitivity and specificity across the full range of activity intensities, as indicated by the overall classification results. This suggests that, while absolute test–retest stability at very high intensities is limited, the upper-arm placement provides a robust and informative signal for classifying activity intensity across everyday PA, capturing both propulsion-related and non-propulsion-related upper-body movements. In practical terms, it appears to offer an optimal compromise between accuracy and usability, particularly when chest placement is not feasible. Test–retest agreement can be considered acceptable, particularly for SB to MPA, while stability at very vigorous intensities was limited.

### Strengths and limitations

A strength of this study is, addressing an important gap in research on accurately measuring energy expenditure, as well as the use of a gold-standard metabolic cart (MetaMax 3B) in a subsample to directly validate energy expenditure estimates and PA in CAUW. In this context, we successfully validated the Move4 accelerometers. Additionally, the sensors were determined activity intensity cut-off points, validated and tested at three different body positions, demonstrating their applicability across a wide range of scenarios. The activities included in the protocol followed the Youth Compendium of PA [38], which provides a well-established list of activities and their corresponding MET values as well as the Compendium of PA in Adult Wheelchair users [39]. To further validate the appropriateness of these activity selections and their assigned intensity levels, energy expenditure was assessed using portable metabolic cart, confirming that the performed activities fell within the expected intensity ranges.

This approach ensured that the chosen tasks were meaningful and representative of typical energy expenditures. To further guarantee precise execution, a research assistant actively led and participated in the exercises, which was particularly important for maintaining the defined pace during wheeling. Together, these methodological choices strengthen the validity and reliability of our measurements. Although some variability between measurement occasions was observed, particularly at higher intensity thresholds, overall agreement was acceptable and supports the practical applicability of the derived cut-off points.

Despite the strengths of this study, several limitations should be acknowledged. First, the sample size was relatively small, including only 24 children, of whom only seven were assessed using the portable spirometer to verify MET values. Additionally, some participants experienced difficulties performing certain activities for the full four-minute duration, which required data cleaning and cut-outs during preprocessing. These factors suggest that the validation of activities and the determination of cut-off points should be interpreted with caution. Variability in fitness levels and weight status among participants could have influenced heart rate responses within individual activities. Furthermore, because predefined wheeling speeds rather than self-selected intensities were used, relative exercise intensity may have differed between participants according to individual physical capacity, age, and wheelchair experience. While this standardized approach was necessary to ensure comparability for accelerometer cut-off point determination, it may limit transferability to free-living activity patterns. In addition, heart rate responses during higher-intensity activities may have been influenced by the sequential structure of the protocol and relatively short recovery periods between activities. Although recovery phases were included, residual cardiovascular activation may have contributed to elevated heart rate values, particularly during higher intensity tasks. Therefore, heart rate findings should be interpreted cautiously and considered supportive rather than definitive criterion measures. Nevertheless, the data indicated good validity of both the activities and the corresponding MET values. Future studies could aim to include multiple activities for each intensity level to enhance generalizability and robustness of the findings.

### Implications for future research

The present study provides initial accelerometer-based cut-off points for PA intensity in CAUW. While these findings contribute to improving device-based assessment in this specific population, they also highlight several important directions for future research. First, the observed differences in cut-off values across sensor positions (wrist, upper arm, chest) underline the relevance of placement-specific calibration. This has implications not only for CAUW but also for other populations with movement limitations, such as individuals using walkers, crutches, or other assistive devices. In these groups, movement patterns differ substantially from those of ambulatory populations, suggesting that accelerometer thresholds derived from walking-based samples may not be directly transferable. Future studies should therefore examine whether similar calibration approaches can be applied to other mobility-impaired populations and whether population-specific cut-off points are required to ensure valid intensity classification. Second, our findings raise important considerations for inclusive PA research. In inclusive study settings, where participants with and without mobility impairments are assessed simultaneously, the use of different sensor positions or population-specific cut-off points may complicate direct comparisons of PA outcomes. Future research could systematically investigate whether harmonized measurement strategies, such as parallel use of multiple sensor positions or cross-calibrated cut-off points, allow for meaningful comparisons across heterogeneous samples. Such research would require dedicated study designs explicitly aimed at testing comparability between groups rather than assuming transferability of thresholds. Third, the activity protocol used in the present study focused exclusively on wheelchair-based movements. However, previous research and qualitative reports suggest that some children who use wheelchairs may also engage in PA outside the wheelchair (e.g., sitting or moving on the floor) [61]. Including such activities in future calibration protocols may improve ecological validity and better reflect real-life movement behaviour in this population. Extending calibration procedures beyond wheelchair propulsion could help capture the full spectrum of PA patterns relevant to children with mobility impairments. Finally, future studies should include more diverse samples with respect to age, functional abilities, and mobility modes. Comparative studies involving children who can walk, CAUW, and those who alternate between mobility modes could help disentangle the influence of movement modality from physiological effort. Such approaches would contribute to a more comprehensive understanding of how accelerometer-derived metrics relate to PA intensity across different populations and movement contexts.

## Conclusion

This study provides the first calibration and validation of the Move4 accelerometer for the assessment of PA intensity in CAUW. Using a multi-criterion approach combining accelerometery, heart rate, and MET values measured with metabolic cart, the study established population- and placement-specific cut-off points for SB, LPA, MPA and VPA. The results demonstrate that both MAD and MAI metrics are suitable for intensity classification in this population, with the upper-arm placement showing the best balance between sensitivity and specificity. Overall, these findings represent an important step toward improving the validity of device-based PA measurement in CAUW and provide a methodological foundation for more accurate and comparable assessments in adaptive PA research.

## Data Availability

The datasets used and/or analysed during the current study are available from the corresponding author on reasonable request.
